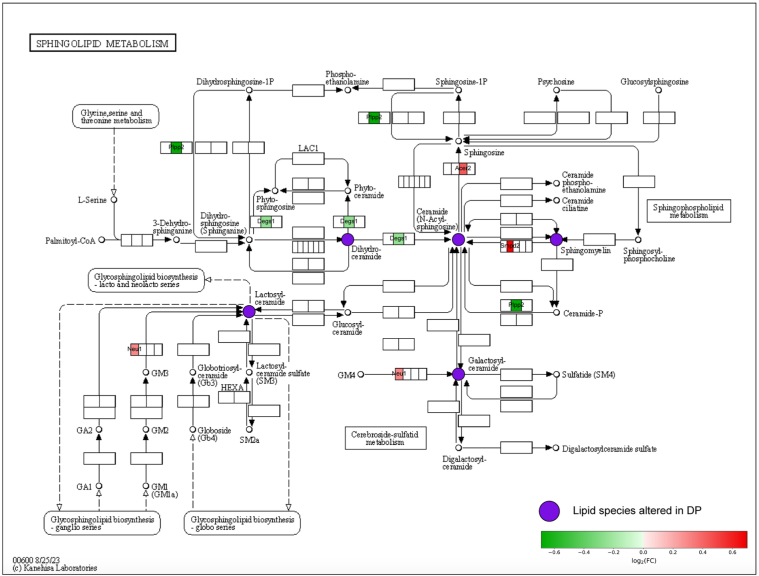# Altered liver lipidome markedly overlaps with human plasma lipids at diabetes risk and reveals adipose-liver interaction

**DOI:** 10.1016/j.jlr.2025.100767

**Published:** 2025-03-04

**Authors:** Ratika Sehgal, Markus Jähnert, Michail Lazaratos, Thilo Speckmann, Fabian Schumacher, Burkhard Kleuser, Meriem Ouni, Wenke Jonas, Annette Schürmann

**Affiliations:** 1 Department of Experimental Diabetology, German Institute of Human Nutrition, Potsdam-Rehbruecke, Nuthetal, Germany; 2 German Center for Diabetes Research (DZD), München-Neuherberg, Germany; 3 Division of Endocrinology and Diabetology, Department of Internal Medicine 1, University Hospital Ulm, Ulm, Germany; 4 Freie Universität Berlin, Institute of Pharmacy, Berlin, Germany; 5 Institute of Nutritional Science, University of Potsdam, Nuthetal, Germany

**Keywords:** lipid species, type 2 diabetes, obesity, New Zealand obese mice, diabetes-prone/resistant

## Abstract

Present study explores the role of liver lipidome in driving T2D-associated metabolic changes. Elevated liver triacylglycerols, reduced PUFAs, and 86 differentially abundant lipid species were identified in diabetes-prone mice. Of these altered lipid species, 82 markedly overlap with human plasma lipids associated with T2D/CVD risk. Pathway enrichment highlighted sphingolipid metabolism, however, only five of all genes involved in the pathway were differentially expressed in the liver. Interestingly, overlap with adipose tissue transcriptome was much higher (57 genes), pointing toward an active adipose-liver interaction. Next, the integration of liver lipidome and transcriptome identified strongly correlated lipid-gene networks highlighting ceramide [Cer(22:0)], dihydroceramide(24:1), and triacylglycerol(58:6) playing a central role in transcriptional regulation. Putative molecular targets of Cer(22:0) were altered (*Cyp3a44*, *Tgf-β1*) in primary mouse hepatocytes treated with Cer(22:0). Early alteration of liver lipidome markedly depends on adipose tissue expression pattern and provides substantial evidence linking early liver lipidome alterations and risk of T2D.

Over the past decades, there has been an unprecedented surge in the global prevalence of obesity and T2D, largely attributed to poor lifestyle choices (1). According to the recent World Health Organization factsheet, more adults across the globe are overnourished (∼1.9 billion) than undernourished (∼462 million) and obesity is a crucial risk factor for the development of insulin resistance (IR), a hallmark of T2D (2). However, this association of obesity, IR, and T2D is highly variable in the general population, resulting in the presentation of heterogeneous clinical phenotypes (3, 4). Of note, nearly two-thirds of individuals with obesity tend to be normoglycemic and maintain a favorable lipid profile for most of their lifespan (5).

Lipids are complex amphiphilic biomolecules that are not only responsible for maintaining cellular integrity but are also perceived as movers and packers of energy across cells and tissues (6). Recent advancements in the field of lipidomics have made it possible to capture tissue-specific high-resolution qualitative and quantitative snapshots of lipid profiles related to various metabolic diseases (7, 8). Numerous studies in mice and humans have identified multiple lipid species as potential prognostic biomarkers for obesity and T2D, especially sphingolipids and phospholipids (8, 9, 10, 11). However, our understanding of the lipidome alterations and the molecular mechanisms of these lipid species that are crucial for the development of T2D is still incomplete.

The liver lipidome is a good surrogate for exploring early metabolic changes as it is strongly associated with the circulating lipidome when compared to other metabolically active tissues (11, 12, 13, 14, 15). For instance, a study in C57BL/6 male mice fed with a high-fat diet (HFD) over 14 weeks reported that nearly 60% of the altered liver lipids were also altered in serum, for example, phosphatidylcholines (PCs) were reduced and specific plasmalogens were increased (11). A similar correlation pattern for many lipid species was also reported in obese women without T2D (13). Moreover, as adipose tissue and liver are important regulators of whole-body energy homeostasis and adipose tissue heavily contributes to the liver lipid influx (16), investigating early alterations in the liver lipidome linked with T2D could also shed light on the adipose tissue-liver interaction.

The present study aimed to identify early changes in the repertoire of liver lipid species linked with the development of T2D using genetically identical mice differing in their T2D susceptibility. To translate the current findings to humans, the altered liver lipid species in mice were screened for altered circulating levels in human plasma, which are associated with the risk of developing T2D or CVDs. Furthermore, the relationship between the lipid species and the phenotypic traits was assessed in mice. An integrative approach was used to uncover the underlying molecular mechanisms linked with specific liver lipid species, by merging the liver lipidome and liver or white adipose tissue transcriptome profiles of diabetes-prone (DP) and diabetes-resistant (DR) mice. Most significant liver lipid-gene correlations identified by an in silico approach were further validated in vitro using primary mouse hepatocytes.

## Materials and Methods

### Experimental animal study design

The animal study protocol and experimental design were detailed previously (17, 18). Briefly, female New Zealand obese (NZO) mice were phenotypically characterized as either DP or DR after 5 weeks of HFD feeding. The risk criteria were based on fasting blood glucose levels and liver fat content at week 10. The DR group remains normoglycemic for the majority of their lifespan, while the DP group develops hyperglycemia (∼14–15 weeks old) when continued on an HFD (17, 18). For the current study, the liver and gonadal white adipose tissue (gWAT) of 10-week-old DP and DR mice were harvested. All procedures involving animals were approved by the DIfE animal welfare committee and local authorities (2347-28-2014, Landesamt für Umwelt, Gesundheit und Verbraucherschutz, Brandenburg, Germany).

### Tissue processing, RNA sequencing, and immunoblotting

The liver and gWAT were collected from a larger animal cohort (18) along with the liver transcriptomics data (18). For gWAT, RNA was extracted using a miRNeasy kit (Qiagen, Germany) as per the manufacturer’s instructions and quantified using 2100 Bioanalyzer (Agilent Technologies, Germany). The RNA samples were sequenced by BGI (Shenzen, China) using BGISEQ. Raw sequencing reads after adapter trimming using Trimmgalorev0.6.5 were aligned to reference genome GRCm38.p6_Ensemble100 via STARv2.7.4a, followed by normalized transcript abundance assessment using STRINGTIEv2.1.3b. Differential expression analysis was done using DESeq2v1.34.0 in R (v.4.1.2). Liver tissue samples were used for the Western blot analysis and a total of 20 μg of protein was separated by SDS-PAGE and transferred onto a nitrocellulose membrane. Blocking was performed using 5% skimmed milk powder in tris buffered saline with tween 20. The primary antibody fatty acid desaturase 1 (FADS1) (1M8L2) (Thermo Fisher Scientific, MA5-42587) and stearoyl-CoA 9-desaturase (SCD1) (E-8) (Santa Cruz, sc-515844) was applied at a 1:1000 dilution in 5% skimmed milk powder. An appropriate secondary antibody (anti-mouse for SCD1 and anti-rabbit for FADS1) was used for detection, which was performed using fluorescence-based imaging on a LICOR system.

### Targeted liver lipidomics

Targeted complex liver lipidomics analysis was performed by Metabolon (Morrisville, NC) using the Metabolon TrueMass® complex lipid panel. From the liver samples, the lipid fraction was extracted by overnight digestion with dichloromethane:methanol at 4°C. The supernatants were collected and subjected to a modified Bligh–Dyer extraction using methanol/water/dichloromethane. The extraction was done in the presence of deuterated internal standards, and for the acquisition of the data, sample extracts were dried under nitrogen and reconstituted in a dichloromethane:methanol solution containing ammonium acetate. The extracts were transferred to vials for infusion-MS analysis that was performed on a Shimadzu LC with nano PEEK tubing and the Sciex SelexIon®-5500 QTRAP with a total of more than 1100 multireaction monitoring modes. Both positive and negative mode electrospray was analyzed for all the samples. Individual lipid species were quantified as the ratio of signal intensity of a specific target molecule to its corresponding internal standard and multiplied by the concentration of the spiked internal standard.

The complex lipidome panel analysis resulted in the identification of 941 individual lipid species belonging to 14 distinct lipid classes (cholesteryl esters [CEs], monoacylglycerols, ceramides [Cers], dihydroceramides [dCers], lactosylceramides [lCers], hexosylceramides [hCers], sphingomyelins [SMs], lysophosphatidylethanolamines, lysophosphatidylcholines, diacylglycerols [DAGs], triacylglycerols [TAGs], PCs, phosphatidylethanolamines [PEs], and phosphatidylinositols [PIs]). The total lipid class concentration (nmol/g) or composition (mol%) was calculated by adding the concentrations or compositions of all individual lipid species belonging to one lipid class, respectively. For determination of the fatty acid concentration or composition, all individual fatty acids belonging to a particular lipid class were selected and the sum of the concentrations or compositions was calculated, respectively.

### Adipose tissue and plasma sphingolipids quantification by HPLC-MS/MS

Plasma samples and gWAT homogenates were subjected to lipid extraction using 1.5 ml methanol/chloroform (2:1, v:v) as described (19). The extraction solvent contained C17 Cer and d_31_-C16 SM (d_31_-C16 SM) (both Avanti Polar Lipids, Alabaster) as internal standards. Chromatographic separations were achieved on a 1290 Infinity II HPLC (Agilent Technologies, Waldbronn, Germany) equipped with a Poroshell 120 EC-C8 column (3.0 × 150 mm, 2.7 μm; Agilent Technologies). MS/MS analyses were carried out using a 6495C triple-quadrupole mass spectrometer (Agilent Technologies) operating in the positive ESI mode. Cer and SM were quantified by multiple reaction monitoring (qualifier product ions in parentheses): [M-H_2_O+H]^+^ → *m/z* 264.3 (282.3) for all Cer and [M+H]^+^ → *m/z* 184.1 (86.1) for all SM subspecies (C16, C18, C20, C22, C24, and C24:1) (20). Peak areas of Cer and SM subspecies, as determined with MassHunter Quantitative Analysis software (version 10.1, Agilent Technologies), were normalized to those of the internal standards (C17 Cer or d_31_-C16 SM) followed by external calibration in the range of 1 fmol–50 pmol on column. The determined sphingolipid amounts were expressed as concentration (pmol/20 μl) for plasma or relative to the tissue mass (pmol/mg) for gWAT.

### Lipid pathway enrichment analysis and visualization

The lipid pathway enrichment analysis (LIPEA) was performed using a web-based tool (21). The functional analysis of lipids was based on each lipid class and individual lipid species belonging to the same class were considered as multiple entries. The lipid species abbreviations were converted to Human Metabolome Database as well as LipidMaps IDs and used as an input to the LIPEA tool, where they were mapped to the Kyoto Encyclopedia of Genes and Genomes (KEGG) compound ID for performing KEGG pathway enrichment. A Python-based in-house pipeline was used to visualize the differentially expressed genes and lipid class on KEGG reference maps (22). In summary, an enrichment file in DAVID functional annotation analysis output format was parsed to retrieve the enriched KEGG pathways and genes per pathway. The elements (orthology and compounds) of the pathways were retrieved through the KEGG subpackage of Biopython (23) and modified according to the enrichment analysis results. Differentially expressed genes belonging to a given enriched pathway were colored according to their respective log_2_ fold change, mapped on an automatically scaled color bar. The lipid classes on the reference maps are identified through their compound IDs as encoded in the KGML file that is retrieved through the KEGG REST module. Lipid classes found to be enriched in a given pathway were assigned a fixed single color.

### Integration of lipidomics and transcriptomics data

Integration of lipidomics and transcriptomics data was performed using a holistic approach, wherein all the differentially expressed liver lipid species and transcripts were included in the analysis. Integration was done with the mixOmics package (24), using a multilevel approach (sparse partial least squares analysis) to account for repeated measures. The algorithm selected variables (lipid species) based on importance and relevance to the response variable (transcripts) while promoting sparsity and interpretability with the additional step of reducing overfitting. The cut-off values for the regression coefficient were set to |0.8| and top lipid-gene pairs were shortlisted for further molecular validation.

### Primary mouse hepatocyte culture and effect of ceramide 22:0 treatment

To validate the identified lipid-gene pairs using the bioinformatics approach as detailed above, the hepatocytes from ∼10-week-old chow diet–fed NZO female mice were isolated and treated with Cer d18:1/22:0 (Cer(22:0); Avanti polar lipids, #860501). The animals were anesthetized using isoflurane followed by cervical dislocation, and the abdominal region was disinfected. The inferior vena cava was then cannulated and perfused (8 ml/min for 10 min) with 30 ml of perifusion buffer (HBSS w/o Mg^2+^ and Ca^2+^ + 1 mM EGTA). The perifusion buffer was then replaced with the 30 ml of digestion buffer (HBSS with NaHCO_3_ + Liberase™) or until the liver was sufficiently digested. The liver was then dissected and transferred to a sterile dish with wash buffer (HBSS with NaHCO_3_ + 0.25% FBS + 5 mM glucose). The liver homogenate was filtered over the sterile nylon gauze followed by centrifugation at 40 *g*, 2 min, 16°C. The supernatant was carefully removed and the pellet was resuspended in 50 ml wash buffer and centrifuged (40 *g*, 2 min, 16°C). Next, the supernatant was carefully removed and Percoll-based separation was performed by adding Percoll (13.9 ml Percoll + 2.1 ml 10X PBS) to the cell suspension and centrifuged at 350 *g*, 7 min, 16°C. The top debris was removed and the cell pellet was resuspended in 5 ml plating media (WilliamsMediumE + 1% (v/v) penicillin/streptomycin + 4% (v/v) FBS + 100 nM dexamethasone + 0.5 nM insulin) for cell counting. The hepatocytes were resuspended in plating media and were plated (100,000 cells/ml) in the mini-petri plates for 3 h, thereafter the plating media was replaced with culture media (plating media without FBS).

Cer(22:0) was reconstituted similarly as described previously (25). Briefly, 5 mg of Cer(22:0) was dissolved in chloroform:CH_3_OH::1:1 and dried down under N_2_ gas. Prewarmed ethanol:dodecane::98:2 (37°C) was added to dried Cer(22:0), the final stock concentration was 2.5 mM, and the solution was further vortexed and incubated at 37°C for 30 min. Further dilutions were done in culture media and a vehicle stock solution was prepared. The working concentration range was selected based on the previous literature (25) and also based on the concentrations in the liver lipidomics data (DR ∼20 μM; DP ∼60 μM). Hence, for the cell viability analysis, the following concentrations were used; 6.25 μM, 12.5 μM, 25 μM, 50 μM, and 100 μM. Cer(22:0) was added to primary hepatocytes the day following hepatocyte isolation, and cell viability was assessed by methylthiazolyldiphenyl-tetrazolium assay (CellTiter96® Aqueous One Solution, Promega). The hepatocytes were lysed after 24 h of exposure to Cer(22:0), total RNA was extracted and quantitative reverse transcription PCR analysis using PrimeTime quantitative PCR primer assays (supplemental Table S1) was performed. Data were normalized to the expression of the endogenous control and for each comparison ΔCt was calculated.

### Western blot analysis

To evaluate the phosphorylation of AKT, Western blot analysis was performed for primary hepatocytes treated with Cer(22:0) for 24 h and stimulated with 10 nM insulin for 8 min. Primary hepatocytes were lysed with RIPA buffer freshly supplemented with phosphatase and proteinase inhibitor cocktails. The protein concentration was determined using BCA assay and 12 μg of protein lysate in 15 μl of lysis buffer was separated using SDS-PAGE, transferred to membrane, and blocked with 5% skim milk. Anti-phosphorylated AKT (Ser473, Cat# 9271L, Cell Signaling Technology) and total-AKT (pan, Cat# 2920, Cell Signaling Technology) antibodies were used, followed by the application of a secondary antibody (goat anti-Mouse IgG (H + L), DyLight 680, Cat# 35519, and goat anti-Rabbit IgG (H + L), DyLight 800 4X PEG, Cat# SA5-35571, Thermo Fisher Scientific). Signals were detected using LICOR and Image Studio Lite Ver5.2 was used for the densitometric analysis.

### Quantification and statistical analysis

For the present study, the composition (mol%) of each lipid species was used to calculate the fold change in DP versus DR group for lipid classes, total fatty acid composition profiles, estimated enzyme activity, and unpaired Student's *t* test with Welch’s correction was used to test for statistical significance. For the differential abundance analysis, the data was log-transformed and Z-scaled before performing the Student's unpaired *t* test with Welch’s correction. For all the above-mentioned analyses, *P* < 0.05 was considered statistically significant and all data were presented as mean ± SD. The Spearman correlation test was performed to check the correlation between the lipid species and the phenotypes. The *P* value (*P*) was adjusted (AdjP) for multiple comparisons using the Benjamini–Hochberg method. The LIPEA tool was used for lipid pathway enrichment and *P* < 0.05 was considered statistically significant. For experiments with primary hepatocytes, two-way ANOVA followed by Bonferroni's posthoc test was performed and *P* < 0.05 was considered significant (GraphPad Prism 10).

## Results

### Altered abundance of liver lipid classes in diabetes-susceptible NZO mice

The present study aimed to capture the liver lipid signature of DP and DR mice with a main focus on complex and biologically active lipid species. As both the groups consumed the same diet (60% HFD), it is reasonable to assume that changes in liver lipidome resulted from underlying metabolic alterations. Using the targeted complex lipidomics panel, a total of 941 lipid species categorized into 14 distinct lipid classes were measured (DP, n = 5; DR, n = 4). The lipid classes were further grouped into four categories based on their chemical composition, that is, glycerolipids, phospholipids, sphingolipids, and sterols (Table 1). The targeted lipidome analysis provides both quantitative (nmol/g) and qualitative (mol%) measurements for each lipid species belonging to a particular lipid class as detailed in Table 1. These lipid species, when grouped into lipid classes show that in contrast to TAGs, all other lipid classes were found to be reduced in the DP mice livers compared to DR. Particularly, the proportions of PCs, hCers, lCers), and SMs were reduced in DP mice compared to DR. However, for all further analyses, relative lipid composition (mol%) was considered, as the changes in the overall liver lipid profile were better reflected by altered lipid species proportions rather than concentrations.

**Table 1:** Altered liver lipidome in diabetes-susceptible NZO mice

Lipid Class	Concentration (nmol/g)		Composition (mol%)	
	DR	DP	DR	DP
Glycerolipids				
Monoacylglycerols	247.856 ± 273.929	94.562 ± 18.807	0.324 ± 0.292	0.103 ± 0.016
Diacylglycerols	870.435 ± 103.443	1138.429 ± 302.127	1.358 ± 0.146	1.231 ± 0.242
Triacylglycerols	**30973.435 ± 9680.628**	**56344.756 ± 10958.290**	**46.556 ± 5.098**	**60.835 ± 5.441**
Phospholipids				
Phosphatidylcholines	15564.900 ± 1055.606	15146.814 ± 499.206	**24.509 ± 3.516**	**16.761 ± 2.227**
Phosphatidylethanolamines	11223.006 ± 1847.156	11969.704 ± 1166.835	**17.335 ± 1.119**	**13.300 ± 2.487**
Phosphatidylinositols	228.318 ± 39.802	226.578 ± 17.913	**0.353 ± 0.040**	**0.252 ± 0.047**
Lysophosphatidylethanolamines	**49.359 ± 7.370**	**59.526 ± 9.025**	0.077 ± 0.014	0.067 ± 0.017
Lysophosphatidylcholines	560.007 ± 55.580	562.114 ± 48.455	**0.882 ± 0.145**	**0.625 ± 0.116**
Sphingolipids				
Ceramides	76.218 ± 6.511	84.701 ± 7.048	**0.120 ± 0.017**	**0.094 ± 0.013**
Dihydroceramides	**16.239 ± 0.693**	**18.841 ± 1.968**	0.026 ± 0.004	0.021 ± 0.004
Lactosylceramides	2.659 ± 0.165	2.582 ± 0.323	**0.004 ± 0.001**	**0.003 ± 0.000**
Hexosylceramides	**36.980 ± 2.907**	**29.245 ± 4.848**	**0.058 ± 0.008**	**0.033 ± 0.009**
Sphingomyelins	1430.946 ± 160.332	1423.368 ± 56.512	**2.232 ± 0.228**	**1.576 ± 0.224**
Sterols				
Cholesteryl esters	**3976.612 ± 546.948**	**4648.709 ± 693.695**	**6.165 ± 0.405**	**5.101 ± 0.706**

Diabetes prone (DP, n = 5); diabetes resistant (DR, n = 4); NZO, New Zealand obese. Data were shown as mean ± SD; unpaired Student's *t* test with Welch’s correction; *P* < 0.05 (highlighted in bold).

### Liver fatty acid composition reflects an early alteration of PUFA biogenesis in T2D

**Fig. 1:**
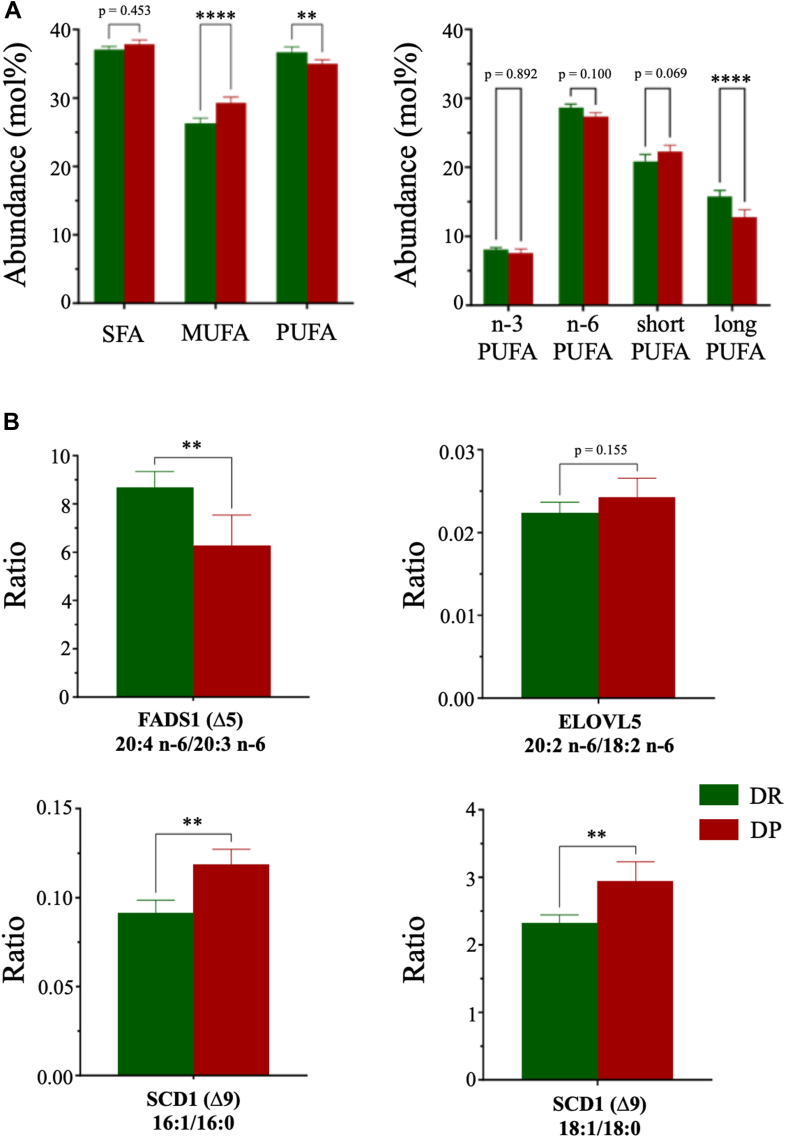
Liver fatty acid composition and the estimated enzyme activities highlight altered PUFA biogenesis in diabetes-susceptible mice. A: The bar plot represents relative abundance (mol%) of saturated fatty acids (SFAs), MUFAs, PUFAs (left), and the abundance of n-3, n-6, short-chain (short), long-chain (long) PUFAs (right). B: Estimated enzyme activities based on product-to-precursor ratios of the fatty acids. Top panel enzymes involved in PUFA biogenesis and the bottom panel enzymes involved in SFA to MUFA conversion. Data shown as mean ± SD; DP (n = 5), DR (n = 4); ∗∗*P* < 0.01, ∗∗∗∗*P* < 0.0001; unpaired *t* test with Welch’s correction. DP, diabetes prone; DR, diabetes resistant.

**Figure S1:**
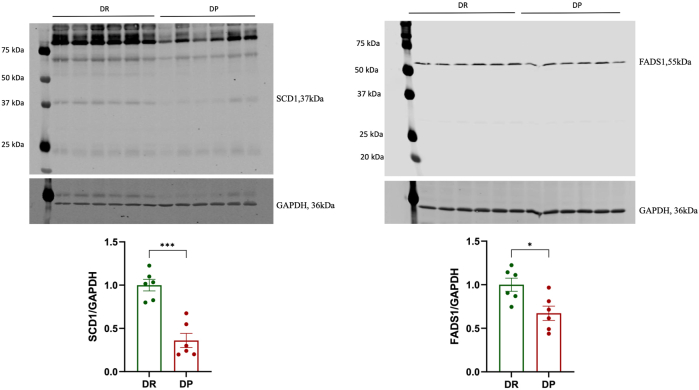


The liver fatty acid composition of the DP mice was found to be altered in comparison to DR mice. Interestingly, there were no changes in the abundance of saturated fatty acids (SFAs), but rather an increase in MUFAs and a significant reduction in PUFAs abundance (Fig. 1A). Further analysis of the PUFAs, indicated a drastic reduction in the long-chain PUFAs with no alterations in n-3, n-6, and short-chain PUFAs (Fig. 1A). Next, the activity of the enzymes involved in the synthesis of long-chain PUFAs was estimated by calculating the product-to-precursor (fatty acids) ratio. The estimated activity of FADS1, a key regulator of the synthesis of long-chain PUFAs, was found to be reduced in the DP liver compared to DR (Fig. 1B). However, no changes were observed in the estimated activity of fatty acid elongase 5. Moreover, the estimated activity of SCD1, involved in the conversion of saturated to unsaturated fatty acids, was found to be increased in DP mice, corroborating with the increased MUFAs abundance (Fig. 1B). Protein abundance of FADS1 enzyme was also found to be significantly reduced in the DP liver compared to DR, supporting an overall reduced liver PUFA synthesis in the early stages of metabolic dysregulation. Contrary to the increased enzyme activity of SCD1 enzyme activity in livers of DP mice, the SCD1 protein levels were found to be reduced drastically compared to DR mice (supplemental Fig. S1).

### Liver lipid species abundance is altered way before the onset of hyperglycemia in DP mice

**Fig. 2:**
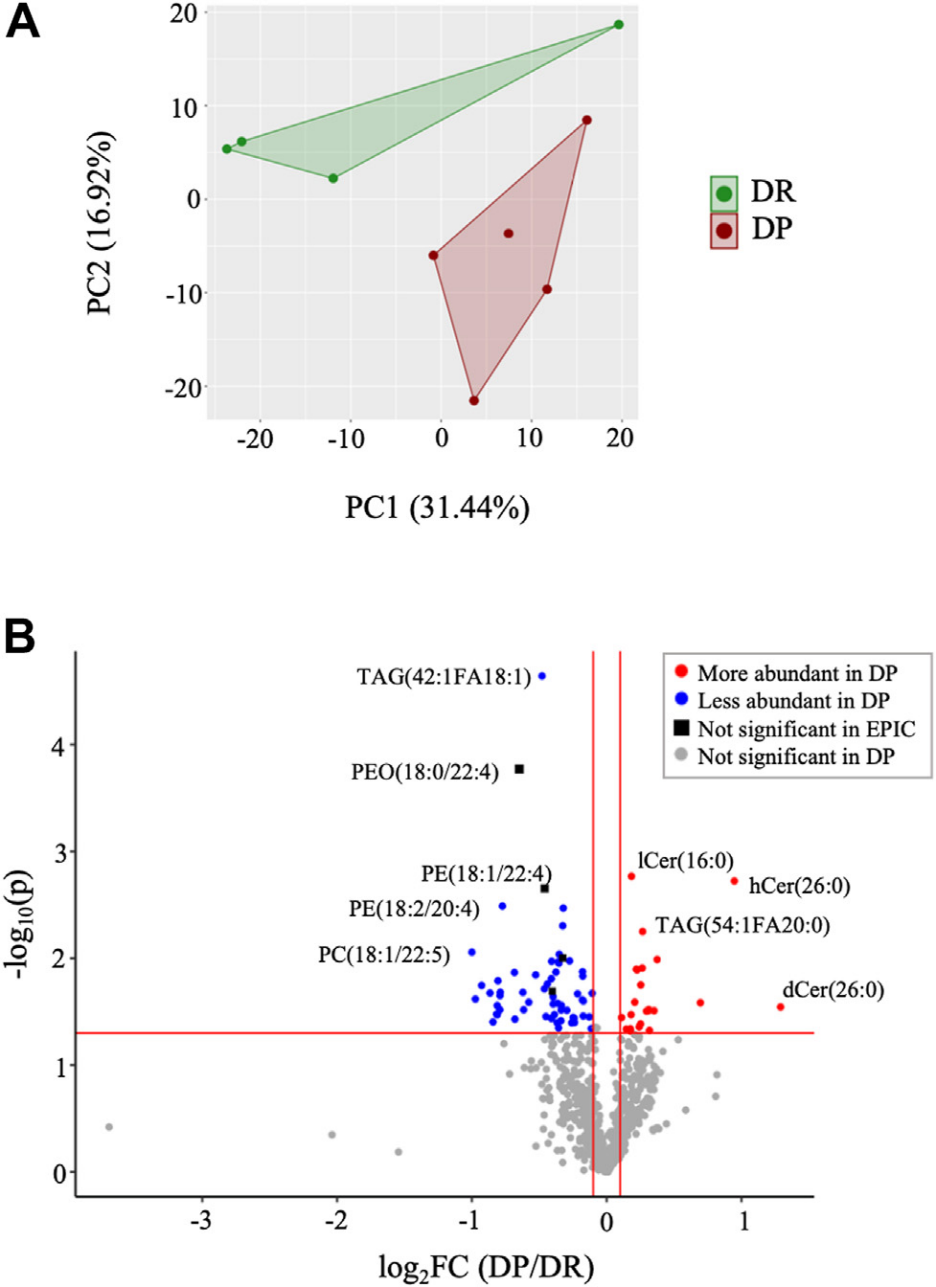
Altered liver lipidome associated with the risk of developing T2D. A: Principal component analysis (PCA) of 812 lipid species (DP, n = 5; DR, n = 4). B: Volcano plot shows differentially abundant lipids. The *x*-axis represents fold change for lipid species, log_2_FC(DP/DR) and the *y*-axis represents −log_10_(*P*); unpaired *t* test with Welch's correction; red dots, increased abundance in DP; blue dots, decreased abundance in DP; and gray dot, *P* > 0.05 in DP versus DR. DP, diabetes prone; DR, diabetes resistant.

Out of all the 914 lipid species that were measured, 812 were detected in all analyzed samples. The principal component analysis using the composition (mol%) of the 812 detected lipid species shows a good separation of the two groups (Fig. 2A). A total of 86 lipid species were differentially abundant (*P* < 0.05) in the liver of DP mice compared to DR (Fig. 2B). Specifically, 62 lipid species were less abundant and 24 lipid species were more abundant in the DP group. The most significant changes were found in the abundance of long-chain TAG (TAG(42:1FA18:1), TAG(54:1FA20:0)), PE(18:1/22:4), phosphatidylethanolamine ether- PEO(18:0/22:4), PE(18:2/20:4)), and ceramide derivatives (lCer(16:0), hCer(26:0), and dCer(26:0)).

### Liver lipidome of NZO mice correlates significantly with phenotypes

**Fig. 3:**
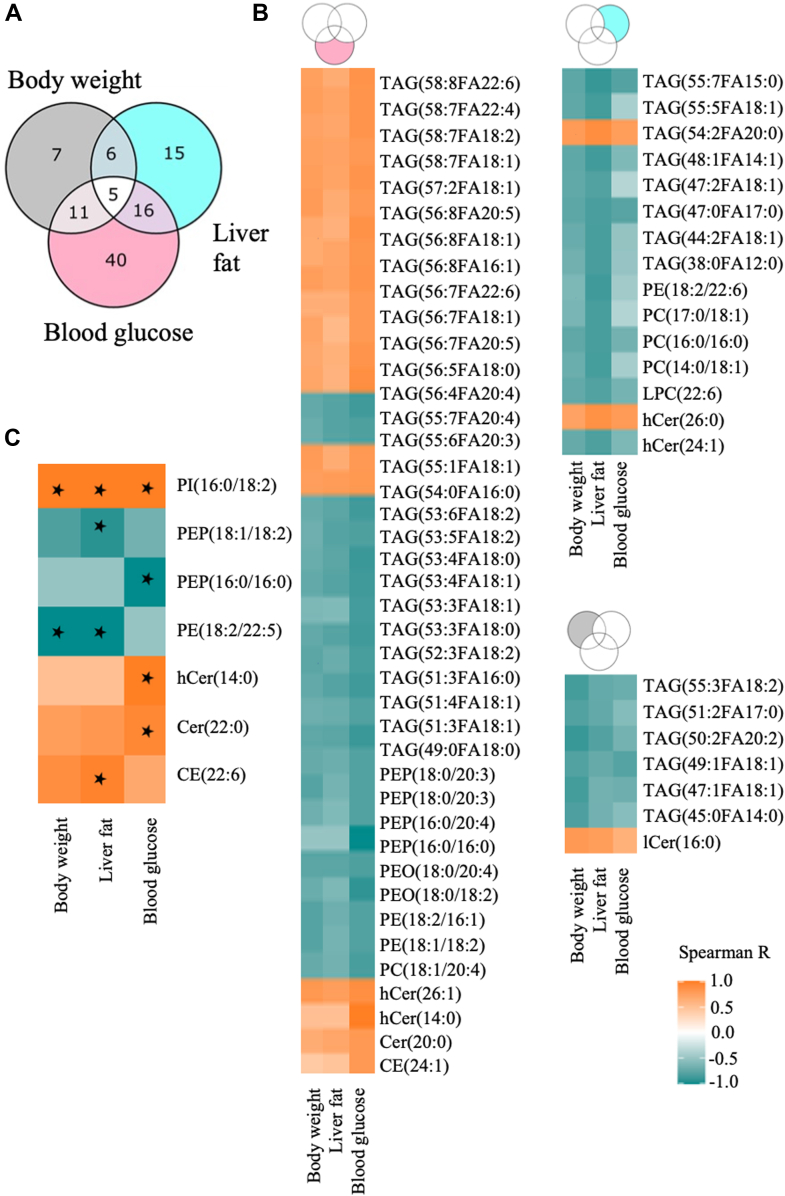
Liver lipid species correlate significantly with T2D-risk-defining phenotypes. A: Venn diagram shows number of lipid species significantly correlated with the indicated phenotype. B: Heat maps for four main arms of the Venn diagram (top of each heat map). *Y*-axis lipid species; *x*-axis phenotypes and all the correlations represented are significant (*P* < 0.05). C: heat map lists the most strongly correlated lipid species with at least one of the phenotypes (Star - AdjP < 0.1). Each cell in the heat map is the Spearman correlation coefficient and the color scale defines the direction of the association for all the heat maps.

To identify the liver lipid species that mirror the phenotypic changes characteristic of DR and DP groups, correlation analysis was performed. The Venn diagram in Figure 3A shows the overlap of the significantly (*P* < 0.05) correlated lipid species with body weight, liver fat content, and blood glucose levels. Overall, the majority of the lipid species correlated exclusively with blood glucose levels (n = 40), followed by liver fat (n = 15) and body weight (n = 7) as shown in the heat maps (Fig. 3B). Moreover, five lipid species were found to be correlated with all three phenotypes (Fig. 3A). Notably, the lipid species that were strongly correlated (AdjP < 0.1) with at least one of the phenotypes were phospholipids, ceramides, and sterols (Fig. 3C).

### Altered sphingolipid biology emphasizes active adipose-liver interaction

To explore the biological relevance of the liver lipidome, pathway analysis was performed for the altered lipid species using the LIPEA online tool. A total of eight pathways were enriched (AdjP < 0.05), of which sphingolipid metabolism and signaling were the top hits (supplemental Table S2). Specifically, ceramides and ceramide derivatives were the main lipid classes in most of the enriched pathways. To check if these changes are also reflected in the expression of genes involved in the same pathway, the liver transcriptome of the DP mice was investigated. The global changes in the liver transcriptome have already been published before (18) and for the current comparison, only the genes involved in sphingolipid metabolism were selected. A total of 55 genes belong to this pathway, and only five genes were differentially expressed (*P* < 0.05) in DP liver (supplemental Fig. S2).

**Fig. 4:**
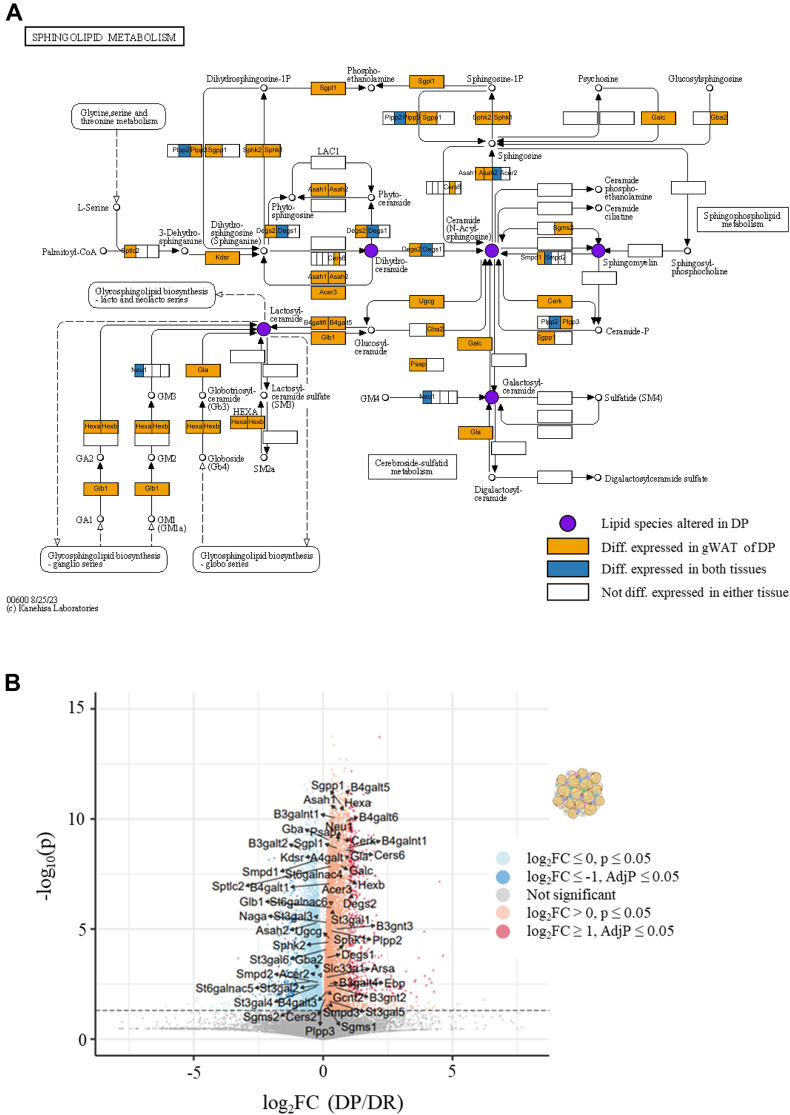
Adipose-liver interaction mediated by sphingolipids altered in DP mice. A: overlap of the lipidomic (liver) and transcriptomic (liver and gWAT) signatures mapping to the sphingolipid metabolism pathway (KEGG - mmu00600). Circles represent lipid species and the rectangles represent genes. B: Volcano plot highlights the genes mapping to the sphingolipid metabolism pathway and subpathways found to be differentially expressed in the gWAT of DP mice. *Y*-axis represents the *P* value with gray dotted line (*P* < 0.05) and *x*-axis represents the fold change. gWAT, gonadal white adipose tissue; KEGG, Kyoto Encyclopedia of Genes and Genomes; DP, diabetes prone; DR, diabetes resistant.

Adipose tissue is one of the major contributors to the liver lipid influx and adipose tissue lipid metabolism is known to be dysregulated in T2D (26). Thus, the expression of these 55 genes was investigated in the gWAT of DP and DR mice. Surprisingly, 31 genes were differentially expressed in the gWAT of DP mice, including the five that were differentially expressed in the liver (Fig. 4). This finding suggests an active adipose-liver interaction contributing to the altered liver lipid profile associated with the risk of developing T2D in the present mouse model. Moreover, when all the genes involved in the sphingolipid metabolism pathway as well as subpathways were combined (n = 216), a total of 57 genes were found to be differentially expressed in gWAT of the DP mice (Fig. 4B).

**Fig. 5:**
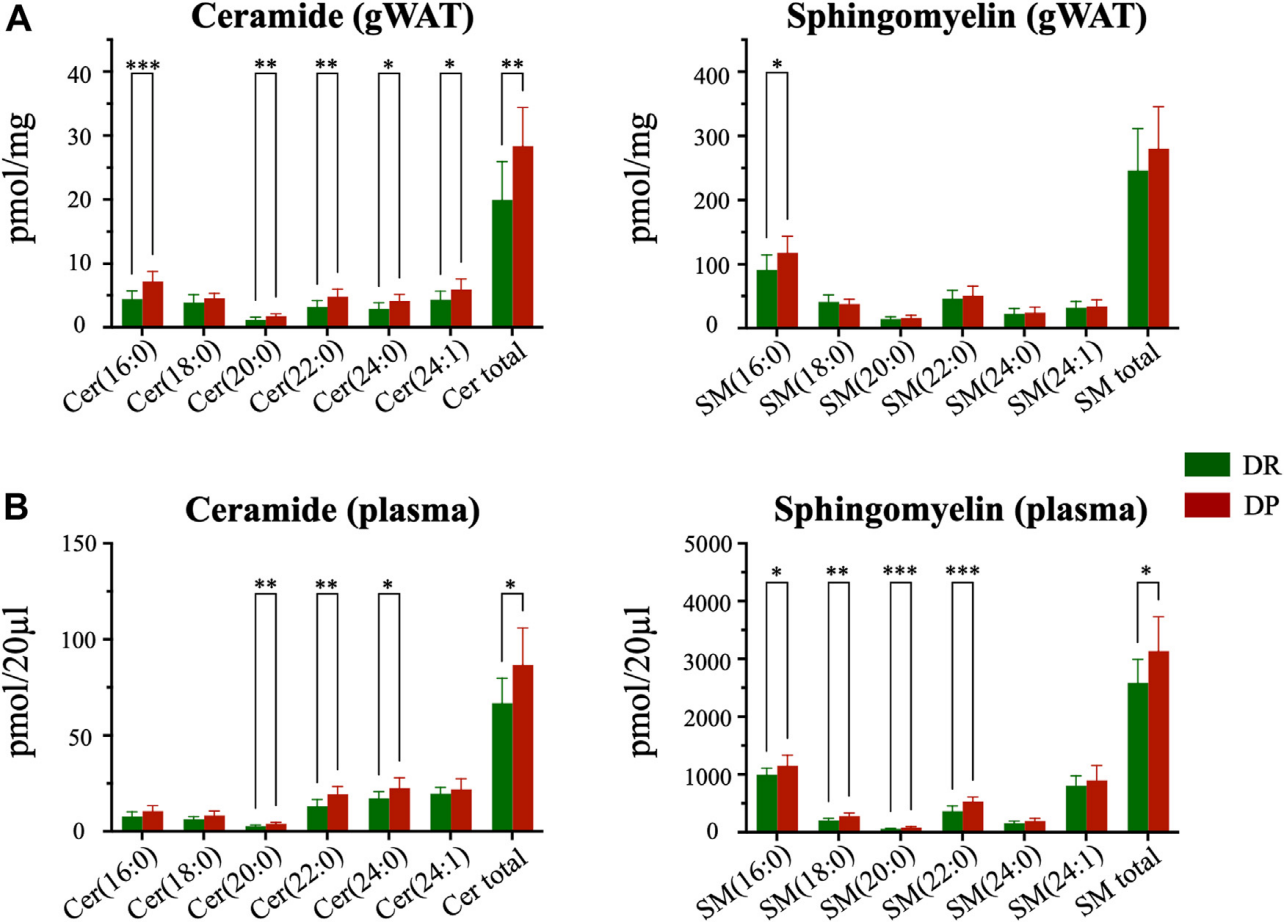
Elevated sphingolipids in both adipose tissue and plasma of DP mice. Lipidomic analysis of ceramide (Cer) and SM species in gonadal white adipose tissue (gWAT) and plasma. A: The top panels display ceramide (left) and SM (right) levels in gWAT, expressed as pmol/mg tissue. B: The bottom panels show ceramide (left) and SM (right) levels in plasma, expressed as pmol/20 μl plasma. Each bar represents mean ± SD. Statistical significance between groups is based on either Mann–Whitney or unpaired *t* test depending on the data normality; ∗*P* < 0.05, ∗∗*P* < 0.01, and ∗∗∗*P* < 0.001. DP, diabetes prone; DR, diabetes resistant; SM, sphigomyelin.

Sphingolipids were also measured in the gWAT as well as in plasma samples of the DP and DR mice to test their role as crucial mediators of early adipose-liver crosstalk. Cer levels for Cer(16:0), Cer(18:0), Cer(20:0), Cer(22:0), Cer(24:0), and Cer(24:1), as well as total Cer content was found to be significantly increased in the gWAT of DP mice (Fig. 5A). Moreover, the plasma Cer(16:0), Cer(20:0), Cer(22:0), Cer(24:0), Cer(24:1), and total ceramide were also significantly elevated in DP mice (Fig. 5B). In addition, SM levels for SM(16:0) and total SM were elevated in both gWAT and plasma. Of note, the plasma levels of SM(18:0), SM(20:0), SM(22:0), SM(24:0), and SM(24:1) were also elevated in mice at risk of developing T2D (Fig. 5B).

### Liver lipidome and transcriptome integration unravels novel lipid-gene correlations

**Fig. 6:**
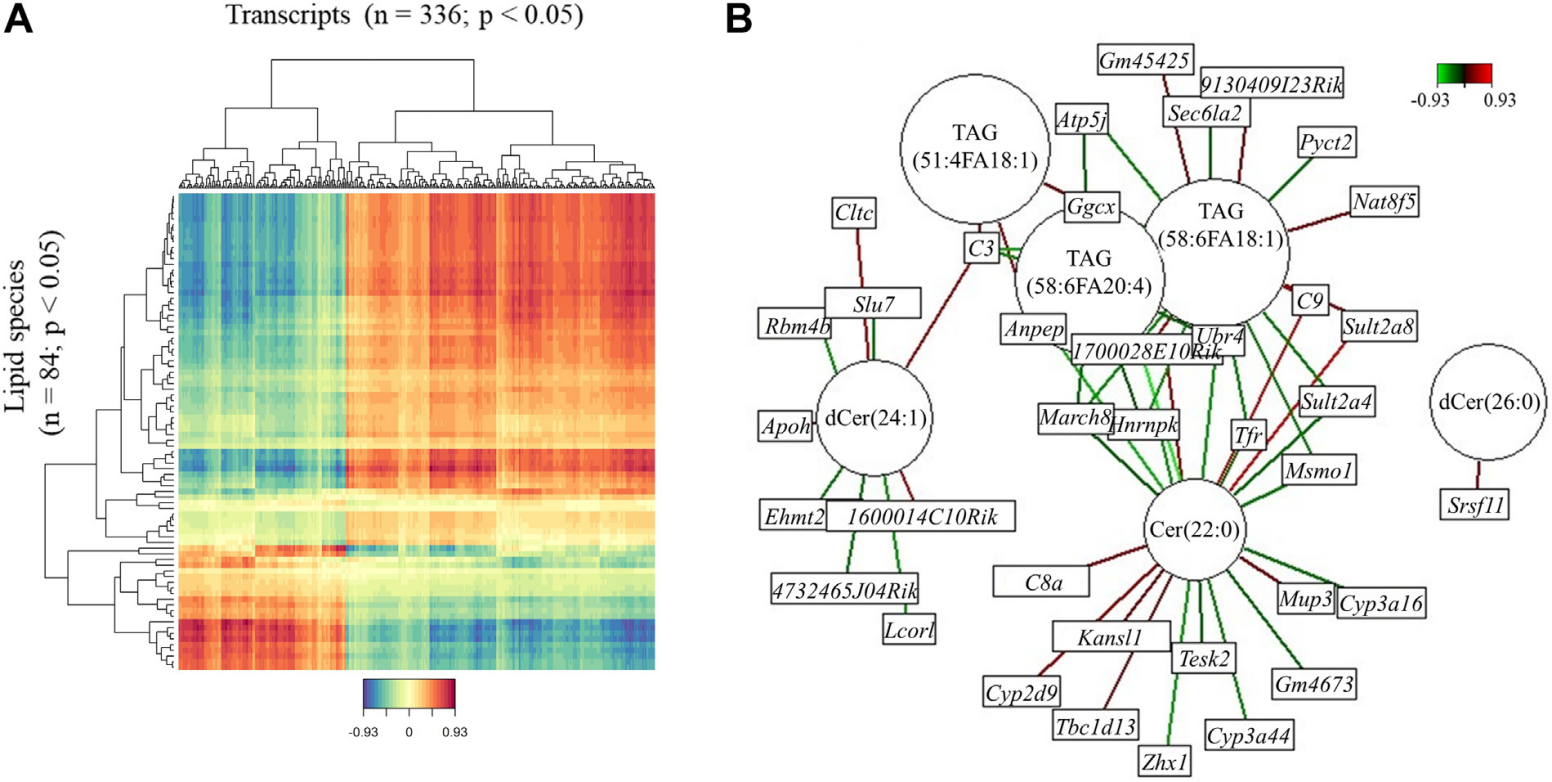
Integration of liver lipidome and transcriptome to decipher novel putative lipid-gene networks. A: Heat map with lipid species on *y*-axis and transcripts on *x*-axis based on the regularized canonical correlation analysis coefficient (DP, n = 6; DR, n = 5). The color key defines the direction of the correlation (blue-negative, red-positive). B: Lipid-gene network (cut-off value of |0.8|). Circles, lipid species; rectangles, transcripts; and lines connecting the lipid species to the transcripts are colored based on the direction (red-positive, green-negative) of the correlation, and the ones highlighted in bold were further evaluated in vitro. DP, diabetes prone; DR, diabetes resistant.

To identify the potential molecular targets of the altered lipid species in the liver, an unsupervised approach of integrating the liver lipidome and liver transcriptome was used. Regularized canonical correlation analysis was performed with differentially abundant lipid species (n = 84, *P* < 0.05) and differentially expressed transcripts (n = 336, log_2_FC > 1.5, *P* < 0.05) using the mixOmics package (24) (Fig. 6A and supplemental Table S3). The most significant lipid-gene correlations included Cer and Cer derivatives (Cer(22:0), dCer(24:1), and dCer(26:0)) and long-chain TAG (TAG(51:4FA18:1), TAG(58:6FA20:4), and TAG(58:6FA18:1)) which were correlated with multiple genes (Fig. 6B and supplemental Table S3).

### Altered liver lipidome in DP mice overlap with human plasma lipidome predictive of T2D

**Fig. 7:**
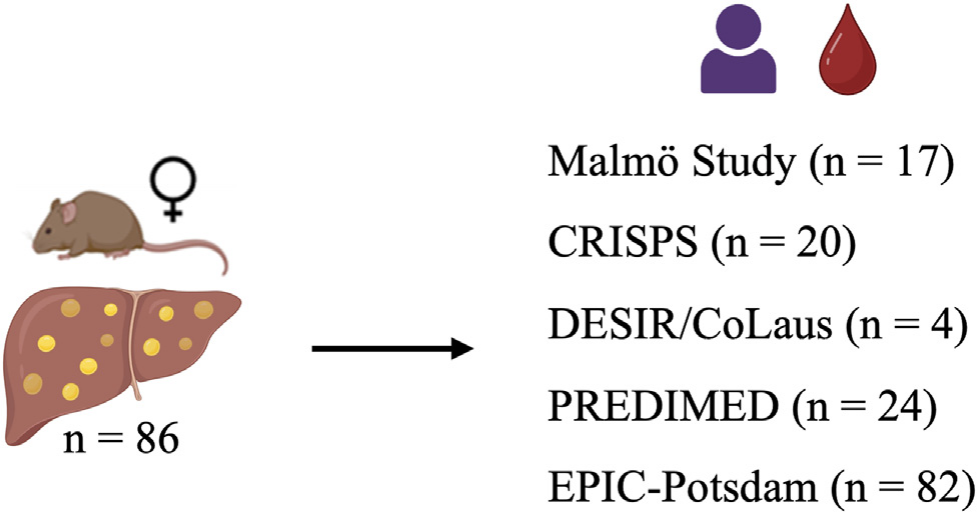
Comparison of altered liver lipidome with human plasma lipidome associated with risk of T2D or CVD. Number of lipid species altered in the liver of mice and also identified to be nominally (*P* < 0.05) associated with the risk of T2D or CVD in human plasma are shown (n). EPIC, European Prospective Investigation into Cancer and Nutrition study (9); PREDIMED, Prevención con Dieta Mediterránea (27); Malmö study, Malmö diet and cancer study (28); CRISPS, Hong Kong Cardiovascular Risk Factor Prevalence Study (29); DESIR/CoLaus, Data from the Epidemiological Study on the Insulin Resistance Syndrome/Cohorte Lausannoise (30).

Of note, a significant proportion of these differentially abundant lipid species have previously been found to be associated with the risk of developing T2D or CVD (Fig. 7). Indeed, 82 of these overlapped with lipid species associated with T2D or CVD risk in human plasma (*P* < 0.05) of individuals from the European Prospective Investigation into Cancer and Nutrition (EPIC) study (9). Moreover, 24 lipid species were also common with the PREDIMED study (27), 17 with the Malmö diet and cancer study (28), and 20 with the Hong Kong Cardiovascular Risk Factor Prevalence Study (29).

### In vitro evaluation of molecular effects of ceramide 22:0 supports in silico findings

**Fig. 8:**
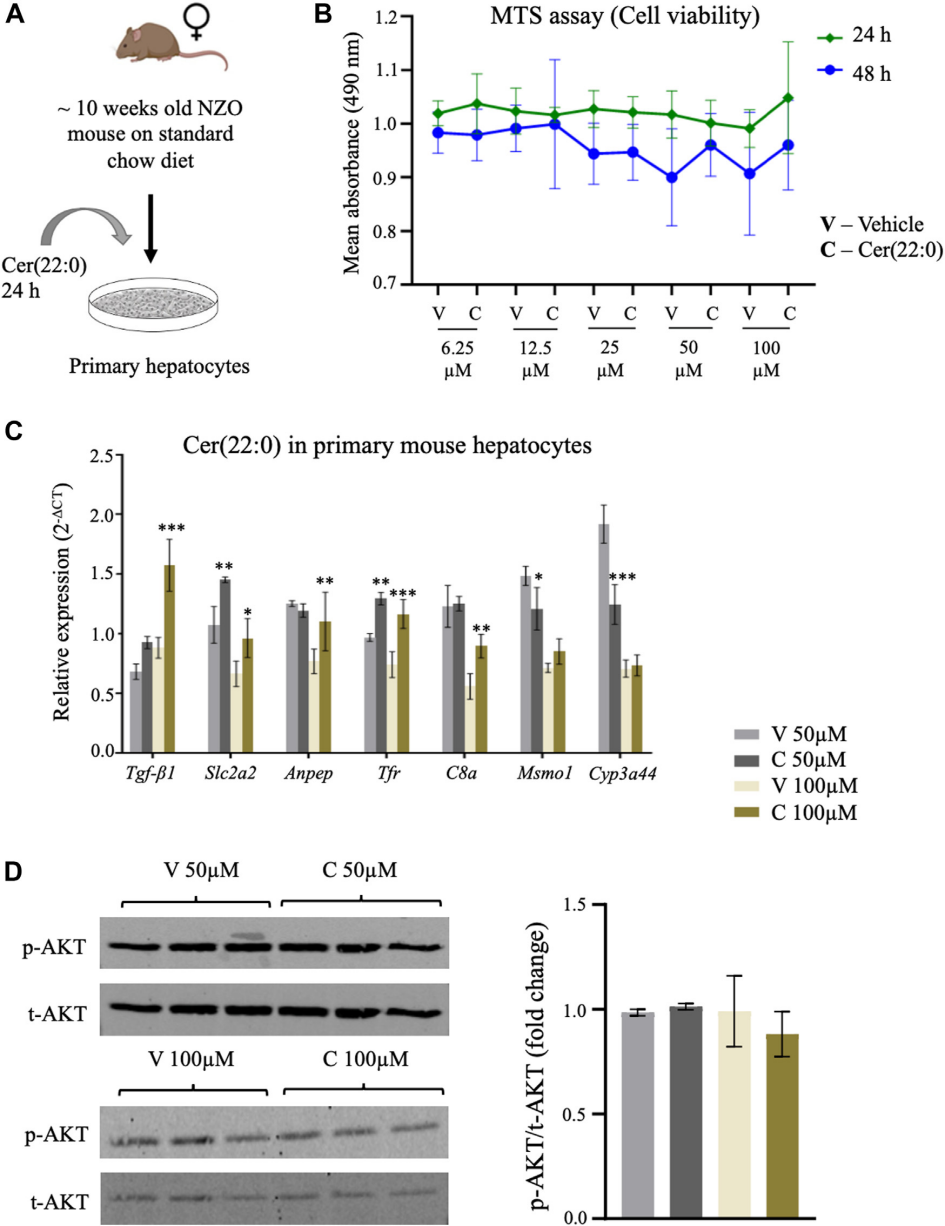
Evaluation of the molecular effects of Cer(22:0). A: Methodology overview. B: Line plot shows the effect of Cer(22:0) on the viability of primary hepatocytes of 10-week-old NZO females with respect to corresponding vehicle control. Data shown as mean ± SD; n = 5/group. C: Bar plot shows gene expression changes after treatment of primary NZO hepatocytes with Cer(22:0) for 24 h. D: Images (cropped) and densitometric quantification of the Western blots for phosphorylated(p)-AKT/total(t)-AKT. Data were shown as mean ± SD; n = 3/group; two-way ANOVA with Bonferroni’s posthoc test for pairwise comparison; ∗*P* < 0.05, ∗∗*P* < 0.01, and ∗∗∗*P* < 0.001. Cer, ceramide; NZO, New Zealand obese.

Based on the in silico findings, Cer(22:0) was shortlisted as the most promising candidate for further molecular validation. Primary hepatocytes from standard chow-fed female NZO mice (10-week-old) cultured in the medium containing up to 100 μM Cer(22:0) showed no significant change in cell viability assessed via methylthiazolyldiphenyl-tetrazolium assay (Fig. 8A, B). In addition, changes in the expression of genes already known to be affected by ceramides (transforming growth factor beta 1 [*Tgf-β1*] and glucose transporter 2, *Slc2a2*), as well as some of those identified using the in silico approach (Fig. 6B), were evaluated. Treatment with 100 μM Cer(22:0) for 24 h increased both the expression of *Tgf-β1*, a classical marker of inflammation, and *Slc2a2* (Fig. 8C). The change in the expression of these genes confirms that Cer(22:0) was taken up by the primary hepatocytes. Moreover, the expression of transferrin receptor, one of the most strongly correlated genes with Cer(22:0) in the in silico analysis was also found to be increased as a response to Cer(22:0) (Fig. 8C). The expression of cytochrome P450, family 3, subfamily a, polypeptide 44 (*Cyp3a44*), and complement component 8 alpha subunit also changed with Cer(22:0) treatment in the same direction as identified by the in silico approach. However, for both the enzymes, alanyl aminopeptidase and methylsterol monooxygenase 1, the expression does not seem to be strongly affected. Since ceramides are known to be linked to insulin signaling thereby playing an important role in the manifestation of IR and T2D (31), the effect of Cer(22:0) treatment on the phosphorylation of AKT was investigated. However, no changes in the status of phosphorylation of AKT were observed for both concentrations of Cer(22:0) (Fig. 8D).

## Discussion

In the present study, the liver lipidome associated with the risk of developing T2D was characterized in diabetes-susceptible NZO mice. Particularly, an early alteration of liver lipid classes’ abundance, fatty acids composition, PUFA biogenesis, a dysregulated sphingolipid metabolism, and an active adipose-liver interaction were identified in the diabetes-prone (DP) mice compared to diabetes-resistant (DR) mice. Of interest, the majority of the altered liver lipids overlapped with the human plasma lipidome associated with the risk of developing T2D/CVD. Furthermore, hepatic lipid-gene correlations highlighted important molecular targets of lipid species, mainly for Cer(22:0), which was also validated in vitro in primary mouse hepatocytes.

The liver lipid composition in both rodent and human models of T2D has been shown to be the primary factor responsible for adverse metabolic and lipotoxic outcomes (32, 33). In the present study, we identified the liver lipid class composition to be altered in DP mice, with an elevation of TAG and a reduction of all other lipid classes (Table 1). Similar findings have been reported previously, wherein, total liver TAG was found to be increased and PC, PI, and Cer were reduced in HFD-fed mice (11). In another study, out of 12 detected liver lipid classes, only TAG was elevated in mice with non-alcoholic fatty liver disease (34), now named metabolic dysfunction–associated steatotic liver disease (MASLD). On the contrary, no alterations were reported in total liver TAG, DAG, and PE abundance in obese insulin-sensitive (high-starch diet) versus obese insulin-resistant (HFD) mice (35). However, in various human studies, the liver TAG increased with the severity of liver damage and correlated strongly with homeostatic model assessment for insulin resistance (36, 37, 38). Altogether, the specific increase in liver TAG highlights the important role of TAG in initiating severe metabolic dysregulations leading to T2D. However, besides TAG, fatty acids and other lipid species may contribute to IR, impaired liver function, and T2D.

The analysis of the liver total fatty acid composition demonstrated an elevated abundance of MUFAs and reduction of PUFAs (in particular long chain) in the liver of DP mice (Fig. 1). These findings align with a previous study where mice fed with a high-fat/high-sucrose diet were found to have increased levels of MUFAs and reduced levels of PUFAs in the liver (39). Similar changes were found in the plasma of individuals grouped as metabolically unhealthy obese, with no changes in SFAs (40). Likewise, plasma n-6 PUFAs were negatively associated with T2D risk in the EPIC-Potsdam study (41). The liver SFAs were not elevated and long-chain PUFAs were reduced in DP mice as well (Fig. 1), reinstating the physiological and metabolic resemblance of the NZO mouse model to early T2D phenotype in humans. However, it is important to note that as disease severity increases, the SFA levels also increases both in the liver and in circulation (38, 42, 43), indicating that SFAs elevation is not necessarily the causal link in the development of T2D. The changes in fatty acid composition were accompanied by altered estimated enzyme activities, reduced FADS1, and elevated SCD1 (Fig. 1). Evaluation of the protein abundances of the following enzymes in the liver of DP mice further confirmed a reduced PUFA synthesis and a dysregulated lipid metabolism. Reduced SCD1 protein levels might also reflect infiltration of the lipid species into the liver from adipose tissue or circulation. The activity of the rate-limiting enzyme of the de novo lipogenesis (SCD1) and PUFA biogenesis (FADS1) has been identified as crucial hubs for defining fatty acid biogenesis both in mice and humans (43, 44). In fact, reduced liver FADS1 enzyme activity was also inversely correlated with homeostatic model assessment for insulin resistance in individuals with MASLD (45). These results suggest an important link between the altered PUFA biogenesis in the liver and the risk of T2D.

Nearly 10% of the identified liver lipid species were significantly different between DP and DR mice, which are genetically identical (Fig. 2). Some of these lipids have previously been found to be linked with T2D (elevated long-chain TAG, CE(18:0), Cer(16:0), SM(14:0), PE(36:2), Cer(22:0), and reduced SM(34:2), PC(38:6), Cer(24:1), and Cer(24:2)) (14, 46). These early alterations of the liver lipidome might provide clues to lipid-derived metabolic changes in the liver leading to T2D. As the fasting circulating lipidome has been previously shown to be strongly correlated with the liver lipidome (12, 13, 15), we overlapped the altered liver lipidome of DP mice with the human plasma lipidome associated with risk of T2D or CVD (9, 27, 29) (Fig. 7). In EPIC-Potsdam study, a total of 69 lipid species were identified as disease risk-associated (either T2D or CVD, false discovery rate < 0.05) based on targeted plasma lipidome analysis. For the present study, not only 69, but all the plasma lipid species found to be associated (*P* < 0.05, all models) with T2D or CVD risk were screened in DP liver (9) resulting in almost complete overlap with similar direction of alteration. Some of these lipids were also differentially abundant in the plasma of individuals grouped either as insulin-sensitive or insulin-resistant (mainly Cer and phosphatidylethanolamine plasmalogen [PEP]) (47). Long-chain TAGs and ceramides were associated with T2D risk in almost all these studies. Altogether, the liver lipid species that could potentially regulate the metabolic derangements leading to T2D were identified prior to the T2D manifestation indicating that they play an important role in the development and progression of the disease.

The abundance of liver lipid species in DP and DR mice correlated strongly with their phenotypic characteristics and the majority of these correlated with blood glucose levels including TAG, sphingolipids, and phospholipids (Fig. 3). Most of these lipids (CE(24:1), PE(18:1/18:2), PEO(18:0/20:4), PEP(16:0/20:4), SM(14:0), TAG(>50), PC(18:1/20:4), Cer(20:0), Cer(22:0), hCer(14:0), hCer(26:1), PEP(16:0/16:0)) have also been found to be significantly correlated with either fasting insulin or insulin secretion in the plasma of metabolically challenged rodents as well as two human prospective cohorts (30). In general, DAG correlates strongly with body weight and hepatic IR (37), however, only four types of DAG were altered in DP liver, and did not correlate with blood glucose levels. The liver sphingolipids dCer(22:0) and dCer(24:1) are known to be negatively correlated with whole-body insulin sensitivity and hCer(22:0) and lCer(24:0) with liver inflammation (48). Plasma Cer(18:0), Cer(22:0), dCer(20:0), and dCer(22:2) have been found to be positively associated with T2D risk and Cer(16:0) with CVD risk in the EPIC-Potsdam cohort. In addition, plasma Cer(22:0) was not only the strongest T2D risk marker but was also linked to cytokine-induced inflammation (10). We also identified many sphingolipids, including Cer(22:0) and hCer(14:0) to be significantly correlated with blood glucose levels, hinting toward an important role of these lipid species in maintaining insulin sensitivity.

To gain insight into the biological relevance of the altered liver lipidome, pathway enrichment analysis was performed leading to the identification of the sphingolipid metabolism pathway (supplemental Table S2). Sphingolipid and endocannabinoid signaling pathways were also found to be enriched in other obese mice (49), while in a study with obese adolescents PI biosynthesis pathway was enriched (50). Additionally, overrepresentation pathway analysis using KEGG in combination with metabolite set enrichment pathway analysis has been used previously to perform lipid pathway enrichment (51). Even though the lipids involved in the sphingolipid metabolism pathway were altered in DP mice, to our surprise only a few genes were differentially expressed in the liver (supplemental Fig. S2). Finely tuned liver-adipose tissue interaction via sphingolipids leading to hepatic IR has also been proposed using diet-induced MASLD mice (52). A recent animal study demonstrated that the adipose tissue–derived extracellular vesicles are particularly enriched in ceramides, SMs, and phosphatidylglycerols and that their composition changes in the context of obesity (53). These findings prompted us to evaluate the expression of genes mapping to the sphingolipid metabolism pathway in the adipose tissue. We found almost all these genes to be differentially expressed in the gWAT of DP mice (Fig. 4), proposing an active lipid-mediated adipose-liver interaction, which is disrupted before the onset of other metabolic phenotypes in the context of T2D. These findings were further supported by elevated total ceramide and total SM levels in both gWAT and plasma of the DP mice (Fig. 5), way before the onset of hyperglycemia.

To evaluate the impact of affected lipid species on the expression pattern of the liver, we used a more holistic approach, the mixOmics analysis (Fig. 6). In recent studies, the mixOmics package was applied to integrate lipidomics and proteomics data or methylome and proteome data (54, 55). Our analysis identified those lipid species exhibiting maximum interactions with differences in gene expression such as long-chain TAG and Cers (Cer(22:0), dCer(24:1), and dCer(26:0)). These species affect exclusive (e.g., Cer(22:0): *Cyp3a16*) and overlapping transcripts (TAG(51:4FA18:1) and Cer(22:0): alanyl aminopeptidase). Based on previous literature and our results, it is evident that Cer(22:0) abundance is strongly associated with the preservation of insulin action (9, 10, 35). We were further able to validate putative molecular targets of Cer(22:0) in primary NZO mouse hepatocytes (Fig. 8). Ceramides have previously been shown to regulate TGF-β signaling, insulin signaling (Akt/PKB), inflammation (Toll-like receptor 4, CD36), and mitochondrial stress (25, 31, 56). In particular, we found that Cer(22:0) exposure to primary hepatocytes resulted in an unfavorable outcome with increased expression of *Tgf-β1* and component 8 alpha subunit (markers of inflammation) and reduction of methylsterol monooxygenase 1 (enzyme) and *Cyp3a44* (transporter). However, maybe due to the relatively short treatment with Cer(22:0) insulin signaling was not impaired in NZO hepatocytes.

We acknowledge the limitations of the present study. The results are based only on female NZO mice and hence limit their translation. However, the overlap with the EPIC-Potsdam cohort for the altered lipidome signifies that identified changes in the liver of the NZO mice might reflect early milieu independent of biological sex. Additionally, it was not possible to include any lean healthy control group in the comparison and the enzyme activities are based on the estimates hence the results must be inferred accordingly. Both DP and DR groups were obese but with significant differences in body weight. As the liver weight was similar in both groups changes in body weight were considered as a phenotype and not as a causal feature. Overall, this study provides a liver lipid signature representative of early alterations that might trigger metabolic changes linked to T2D.

In conclusion, we propose that an altered liver lipid composition is an early event in the development of T2D and putatively reflects the crucial role of the interaction between adipose tissue and the liver in the manifestation of hyperglycemia. In addition, we identified possible lipid-gene interactions that might link specific lipid species to biological effects in the liver. These in silico findings mandate further mechanistic validation as these could potentially be used as predictive biomarkers for the development of T2D in humans.

## Data availability

The authors confirm that the data supporting the findings of this study are contained within the article and the supplementary information. The raw data from liver RNA sequencing is available with GEO accession number: GSE208630 and all other data are available upon request from the corresponding author.

## Supplemental data

This article contains supplemental data.

## Figures and Tables

**Figure S2:**