# Supplementary material for: Altered liver lipidome markedly overlaps with human plasma lipids at diabetes risk and reveals adipose-liver interaction
Source: J Lipid Res. 2025 Mar 4;66(4):100767. doi: 10.1016/j.jlr.2025.100767 (PMC11997378; doi:10.1016/j.jlr.2025.100767)
Supplement: Supplementary information [file mmc1.docx]

**Supplementary information**

Document S1. Table S1-S3 and Figure legend S1-S2

**Supplementary Table 1 (S1): PrimeTime qPCR assay details (IDT, Life Technologies)**

| **Gene ID** | **PrimeTime qPCR assay ID** |
| --- | --- |
| *Ppia* (endogenous control) | Mm.PT.39a.2.gs |
| *Tgfβ1* | Mm.PT.58.11254750 |
| *Anpep* | Mm.PT.58.9657900 |
| *C8a* | Mm.PT.58.7765129 |
| *Msmo1* | Mm.PT.56a.9733563 |
| *Slc2a2* | Mm.PT.58.13545959 |
| *Tfr* | Mm.PT.39a.22214833 |
| *Cyp3a44* | Mm.PT.58.41628262.g |

**Supplementary Table 2 (S2). Pathway enrichment analysis of the altered lipid species.**

| **Pathway name** | **Pathway lipids (n)** | **p** | **AdjP** |
| --- | --- | --- | --- |
| Sphingolipid metabolism | 21 | <0.0001 | <0.0001 |
| Sphingolipid signaling pathway | 9 | <0.0001 | <0.0001 |
| Necroptosis | 4 | 0.0006 | 0.001 |
| Adipocytokine signaling pathway | 3 | 0.033 | 0.049 |
| Neurotrophin signaling pathway | 3 | 0.033 | 0.049 |
| Insulin resistance | 4 | 0.044 | 0.049 |
| AGE-RAGE signaling pathway in diabetic complications | 2 | 0.022 | 0.049 |
| Leishmaniasis | 4 | 0.044 | 0.049 |

Pathway lipids (n) represent the number of lipid species mapping to each pathway with p-value (p) and p-value adjusted for multiple comparisons using the Benjamini-Hochberg method (AdjP).

**Supplementary Table 3 (S3). Correlation coefficient and p-values between differentially abundant lipid species and differentially expressed genes based on mixOmics.**

Additional file 1

**Supplementary Figure’s legend**

**Supplementary Figure 1: Western blot analysis of SCD1 and FADS1 expression in liver.** Representative western blots and quantification of stearoyl-CoA desaturase 1 (SCD1, 37 kDa) and fatty acid desaturase 1 (FADS1, 55 kDa) protein expression in liver tissue from DR (green) and DP (red) mice. GAPDH (36 kDa) was used as a loading control. The left panel shows the Scd1 blot with its corresponding quantification (SCD1/GAPDH ratio). Scd1 expression is significantly higher in the DR group compared to the DP group (p < 0.001). The right panel presents the Fads1 blot with its quantification (FADS1/GAPDH ratio), showing significantly reduced expression in the DP group (p < 0.05). Data are presented as mean ± standard deviation (SD), with individual data points shown. Statistical significance was determined using the unpaired Student’s t-test.

**Supplementary Figure 2: Overlap of the lipidomic (liver) and transcriptomic (liver) signature mapping to the sphingolipid metabolism pathway (KEGG - mmu00600).** The circles represent lipid species and the rectangles represent genes. The color bar shows the log_2_FC for all the significantly expressed genes with green being downregulated and red being upregulated.
